# Arthrodesis of the Foot or Ankle in Adult Patients with Congenital Clubfoot

**DOI:** 10.7759/cureus.6505

**Published:** 2019-12-29

**Authors:** Thompson Zhuang, Ghida El-Banna, Steve Frick

**Affiliations:** 1 Orthopaedic Surgery, Stanford University, Stanford, USA; 2 Orthopaedics, Stanford University, Stanford, USA

**Keywords:** arthrodesis, congenital clubfoot, osteotomy, pediatric orthopaedics

## Abstract

Background

Although clubfoot that was corrected in childhood rarely recurs in adulthood, persistent deformities or arthritic pain may require further treatment during adulthood. Little evidence exists on the operative procedures utilized in adult clubfoot patients, who were previously treated for congenital clubfoot in childhood, for residual or recurrent deformity or pain.

Objective

The objective of this study is to characterize the types and frequencies of procedures utilized in adult clubfoot patients, who were previously treated for congenital clubfoot in childhood.

Methods

A two-pronged approach was employed to describe the operative procedures used in adult clubfoot patients. First, a literature review of all reported cases of operative treatment in adult clubfoot patients who were previously treated in childhood was performed. Second, an analysis of the operative treatments used in adult patients with a diagnosis of congenital clubfoot was conducted using a large, administrative claims database.

Results

In the literature review, arthrodesis was the most cited operative treatment and reported in four out of the eight studies included. Osteotomies were also reported in the literature. In the database analysis, 94 hindfoot arthrodesis procedures were identified in 73 patients, out of 1,198 adult patients in the database with a diagnosis of congenital clubfoot. Sixty-two patients out of 1,198 adult clubfoot patients received osteotomies. An insufficient number of total ankle arthroplasties were reported for further analysis.

Conclusions

Operative treatment in adult clubfoot patients who were treated for congenital clubfoot in childhood includes hindfoot arthrodesis and osteotomy procedures. Total ankle arthroplasty has not been reported in the literature for these patients.

## Introduction

Congenital clubfoot, also known as congenital talipes equinovarus, is a common birth defect with a prevalence of approximately one in 1,000 live births [1]. Clubfoot that was formerly treated in childhood rarely recurs in adulthood, although persistent deformities or arthritic pain may need treatment in adulthood [2]. Nevertheless, little information exists on clubfoot treatment in adults. Several case studies have described salvage techniques including double and triple arthrodesis procedures for treating neglected clubfoot and late recurring idiopathic clubfoot in the adult patient [2-5]. However, for patients with clubfoot treated in childhood, the need for further surgery in adulthood is not well described in the literature. Parents and patients often have questions about the possible need for surgery as adults for residual or recurrent deformity, or for pain, but little is known about the incidence, indications, or operations used to treat adult clubfoot patients. In addition, medicolegal cases involving young clubfoot patients often calculate estimated lifetime healthcare costs and include future operations such as triple arthrodesis or total ankle replacement when preparing a life care plan cost assessment. For example, lifetime cost assessments, including relevant costs borne in adulthood, for other childhood conditions with medicolegal ramifications have been performed for child maltreatment, hypoxic-ischemic birth injury, and childhood obesity [6-11].

This study evaluates operative treatments used in adult patients with congenital clubfoot treated in childhood by employing two approaches: 1) a thorough literature review of existing cases and 2) an analysis of adult clubfoot patients undergoing ankle or foot surgery in a large administrative claims database.

## Materials and methods

Literature review methods

**Figure 1:**
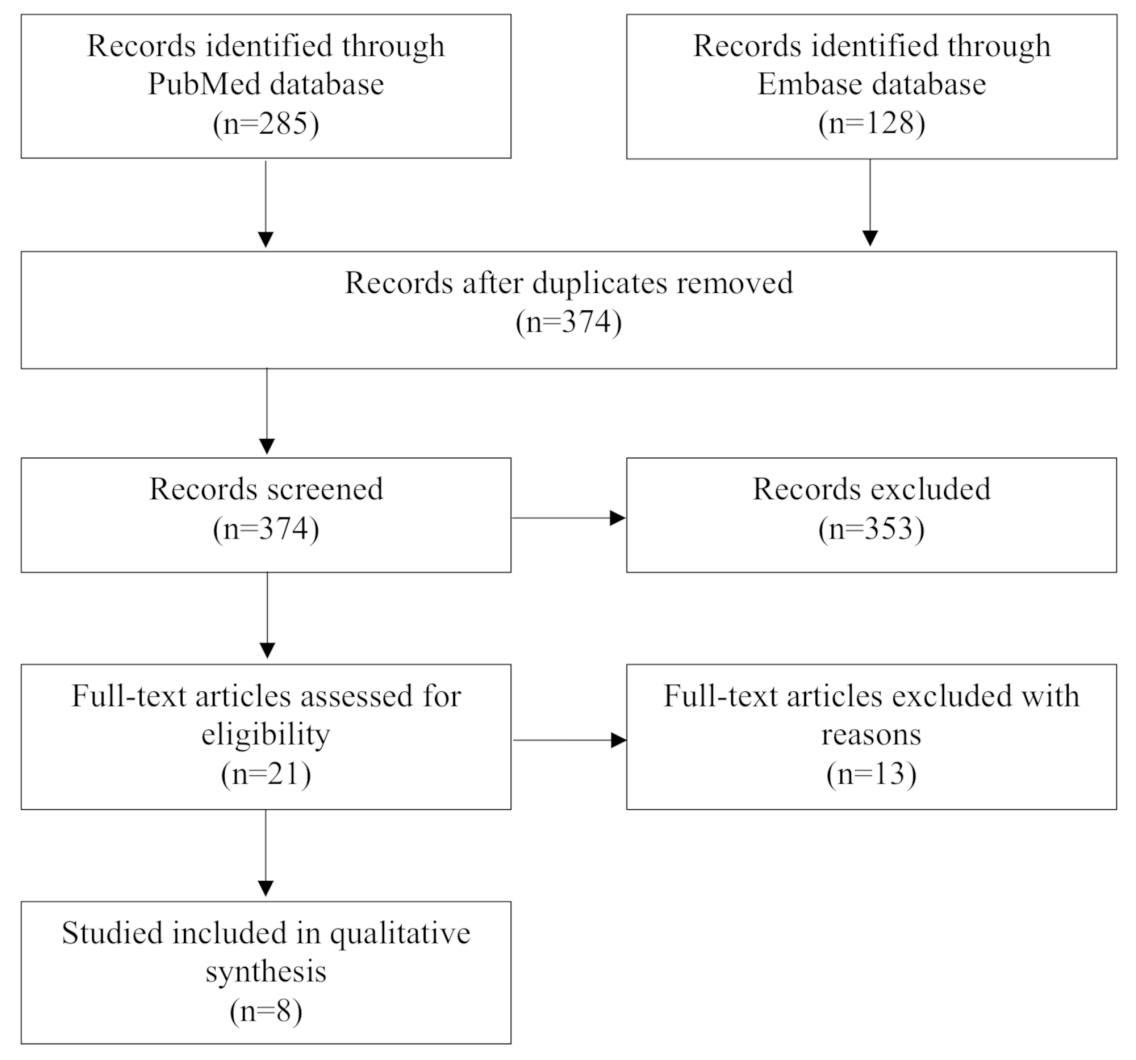
Literature review scheme

A literature search was conducted to capture all articles pertaining to patients with congenital clubfoot, who were treated with either nonoperative or operative methods during infancy and later received operative treatment during adulthood to relieve pain or improve foot position. English-language articles and all years up to September 2019 were included. The search strings used are available in Supplementary Information. The PubMed and Embase databases were searched, which returned 285 and 128 articles in PubMed and Embase, respectively. There were a total of 374 articles after excluding duplicates. Screening abstracts to determine whether the article addressed the operative treatment of previously treated clubfoot in adults led to the exclusion of 353 articles. Twenty-one full-text articles were assessed for eligibility. Included studies reported patients who 1) were diagnosed with congenital, idiopathic clubfoot in the first months after birth, 2) treated for clubfoot during childhood either surgically or non-surgically, and 3) underwent surgical procedure(s) as adults to correct recurrent, residual, or overcorrected clubfoot and foot position or to alleviate foot pain. Only eight articles met these inclusion criteria and were included in the qualitative analysis (Figure 1).

Database analysis methods

Humana’s administrative claims database was analyzed using commercially available PearlDiver software (PearlDiver, Inc.; Colorado Springs, CO). This database comprises patient records from over 25 million members from 2007 to 2017 and includes payer data from both commercial and Medicare Advantage plans. Claims data include dates, diagnoses, procedures, and patient demographics, such as geographic location as defined by the United States Census Bureau. The database contains claims from diverse medical and surgical specialties, including podiatry. This database has been previously used in orthopedic research and its characteristics have been described elsewhere [12]. Because all data were de-identified and anonymous, institutional review board (IRB) approval was not required.

All patient records associated with a diagnosis of congenital talipes equinovarus (congenital clubfoot) were identified using International Classification of Diseases, Ninth or Tenth Revision (ICD-9 or ICD-10) diagnosis codes and included in the analysis (Table 1). Because the database only codes age in five-year buckets, adults were defined as patients at or over 20 years old. All patients under 20 years of age or with a diagnosis of acquired clubfoot were excluded. Demographic information on all patients diagnosed with congenital clubfoot was obtained, including the age, region, and service location associated with each claim. Using Boolean-type search functions, clubfoot patients who underwent mid-tarsal joint arthrodesis, subtalar arthrodesis, ankle arthrodesis, triple arthrodesis, total ankle arthroplasty, or osteotomy procedures were identified using their respective Current Procedural Terminology (CPT) codes (Table 1). Count data are reported for each of the procedures. As they have lower costs and lower morbidity and are relatively infrequent, we did not include soft tissue procedures in our database analysis.

**Table 1:** Definitions of Diagnosis and Procedural Codes used ^†^Includes talonavicular arthrodesis. ICD, International Classification of Diseases; CPT, Current Procedural Terminology

Diagnosis or Procedure	ICD-9/10 or CPT Code
Congenital clubfoot	ICD-9-D-75451, ICD-10-D-Q660
Acquired clubfoot	ICD-9-D-73671, ICD-10-D-M21541, ICD-10-D-M21542, ICD-10-D-M21549
Mid-tarsal joint arthrodesis^†^	CPT-28740
Subtalar arthrodesis	CPT-28725
Ankle arthrodesis	CPT-27870, CPT-29899
Triple arthrodesis	CPT-28715
Total ankle arthroplasty	CPT-27700, CPT-27702
Osteotomy	CPT-28300, CPT-28302, CPT-28304, CPT-28305, CPT-28306, CPT-28307, CPT-28308, CPT-28309

## Results

Literature review

The studies included in this review reported symptomatic adult clubfoot patients with pain, difficulty walking, inability to wear conventional shoes, flatfoot, ossification of the Achilles tendon, and calf muscle hypotrophy. A summary is provided in Table 2. Arthrodesis was the most cited operative treatment (in four out of eight studies). Blitz et al. reported a case of a 22-year-old female with bilateral clubfeet treated with a posteromedial release in childhood [13]. The patient presented with difficulty walking, disfigured feet, and right rearfoot pain. The interposition of the flexor hallucis longus tendon was found intraoperatively, and the patient was successfully treated with triple arthrodesis [13]. Eberhardt et al. presented a series of 21 patients between the ages of 11 and 26 years [14]. These patients presented with flatfeet and pain due to extensive clubfoot surgery in childhood. Six feet were treated with arthrodesis and 19 feet were treated with tarsal osteotomies. Overall, the range of motion did not improve, and 81% of patients were satisfied with the surgery outcomes [14]. Ramseier et al. reported a series of seven patients with unilateral clubfoot in the age group of 18 to 45 years [2]. Childhood treatments included casting, soft tissue release, Achilles tendon lengthening, midfoot arthrodesis, medial release, dorsomedial release, and plantar fascia lengthenings. All were treated with triple arthrodesis. They reported satisfactory outcomes, despite several complications such as residual symptoms, degenerative changes at the ankle in 57% of the patients, and increased ankle arthritis in 67% of the patients. Pain was reduced but not completely alleviated, and ankle motion remained unchanged [2]. Wei et al. reported 16 patients in the age group of 4 to 20 years who received clubfoot release during childhood [15]. Individual patient data were not reported; thus, the number of patients who were at least 18 years old could not be determined. All patients underwent talonavicular arthrodesis for residual midfoot deformities. In addition to arthrodesis, lateral column shortening with a calcaneal wedge osteotomy was performed in eight patients, and two patients required a bone graft to fill a residual gap at the talonavicular fusion site. Fourteen patients were completely satisfied with surgery outcomes, and two patients were partially satisfied. All patients reported pain relief and correction of residual deformities, as reflected in the improvement of the talus-first metatarsal angle [15].

**Table 2:** Summary and characteristics of studies included in the literature review ^†^Age range is 11 to 26 years old, and individual patient information was not available. ^‡^Age range is 4 to 20 years old, and individual patient information was not available. [2], [13-19]

Author(s) & Year	Study Type	Number of Patient(s)	Childhood Treatment(s) (Chronologically)	Clubfoot-related Problem in Adulthood	Adulthood Treatment(s)	Outcomes of Adulthood Treatment(s)
Blitz et al. (2008)	Case Report	1	Posteromedial release	Interposition of the flexor hallucis longus tendon through the subtalar joint	Triple arthrodesis	Satisfactory without complications
Eberhardt et al. (2018)	Prospective	21^†^	Posteromedial release or peritalar release	Hindfoot deformities: one rotational valgus, 14 mild hinge valgus, seven translatory valgus, and three cases with a combination of a rotational and a mild hinge valgus	Tarsal osteotomies in 19 patients, arthrodesis in six patients, and all flatfeet with translator valgus that were initially treated with a tarsal osteotomy required further arthrodesis	No improvement in range of motion, 81% satisfied with surgery outcomes
Ramseier et al. (2007)	Prospective	7	(1) Casting, soft-tissue release, and Achilles tendon lengthening in three cases, (2) Casting, soft-tissue release, and Achilles tendon lengthening; Chopart arthrodesis in one case, (3) Casting, Achilles tendon lengthening, medial release, dorsomedial release, Steindler procedure in two cases, (4) Casting, soft-tissue release in one case	Late recurrent idiopathic clubfoot deformity	Triple arthrodesis	Satisfactory outcomes but degenerative changes at the ankle in 57% of patients, increased ankle arthritis in 67% of patients, pain reduction but not complete alleviation, and unchanged ankle motion
Wei et al. (2000)	Retrospective	16^‡^	Clubfoot release	(1) Triangular navicular, (2) dorsal-lateral subluxation of the talonavicular joint with a secondary forefoot cavovarus deformity and (3) degenerative changes of the talonavicular joint	(1) Talonavicular arthrodesis, (2) additional lateral column shortening with a calcaneal wedge osteotomy in 7 patients, (3) additional bone graft to fill a residual gap at the talonavicular fusion site in two patients	14 patients completely satisfied and two patients partially satisfied due to navicular-cuneiform osteoarthritis in both feet three years after surgery, pain relief, correction of the residual deformities, improved talus-first metatarsal angle
Andjelkov et al. (2018)	Prospective	72	Achilles tenotomy	Undeveloped calves	Calf silicone implant	Excellent results with low complication rates
Knupp et al. (2012)	Prospective	14	(1) Serial casting and manipulation in the first months after birth (2) posteromedial release and release of the talocalcaneal interosseous ligament after relapse	Ankle impingement in patients with overcorrected clubfoot deformity	Supramalleolar tibial osteotomy and additional calcaneal osteotomy or plantar flexion osteotomy in same cases	Low risk of complications, significant pain reduction, and increased ankle motion
Majeed et al. (2015)	Case Report	1	Achilles tendon lengthening	Multiple ossifications of the Achilles tendon	Surgical excision of bony lumps	Satisfactory without complications
Walling (2008)	Case Report	1	Serial casting in the first two years of life	Ankle motion of the equinus position with minus 10° of neutral and an additional 15° of plantar flexion, hindfoot in varus and midfoot abduction	(1) Open tendo-Achilles and flexor digitorum longus lengthening, (2) midfoot capsular releases, (3) calcaneal and first metatarsal dorsiflexion osteotomies	Plantigrade foot with 7° ankle dorsiflexion and correction of the hindfoot varus

Besides arthrodesis, other reported procedures included calf silicone implants, supra-malleolar osteotomy, excision of bony lumps, and tendon lengthening. Andjelkov et al. reported 71 patients with unilateral calf hypoplasia and one patient with bilateral hypoplasia [16]. All had a history of Achilles tenotomy after clubfoot relapse during childhood. These patients presented with calf atrophy, decreased foot size, limited mobility, and decreased tibial length. All patients underwent calf enhancement surgery and reported gratifying aesthetic results [16]. Knupp et al. reported 14 patients in the age group of 19 to 66 years treated with serial manipulations and casting in the first months after birth [17]. All 14 patients had clubfoot relapse that was treated with posteromedial release and a release of the talocalcaneal interosseous ligament during childhood. As adults, these patients presented with calcaneofibular impingement either with or without anterior ankle impingement and painful restriction of the ankle joint. A supra-malleolar tibial osteotomy was performed in all patients. Five patients also had additional calcaneal osteotomy and another five patients had a plantar-flexion osteotomy. All patients reported improved ankle motion, reduced pain, and walking in normal shoes [17]. Majeed et al. reported a case of a 24-year-old male with a history of bilateral clubfeet treated with Achilles tendon lengthening at the age of three [18]. The patient presented to the clinic with several swellings over his right Achilles tendon and was found to have intra-tendinous ossifications. Bony lumps were surgically removed, which resulted in pain reduction [18]. Finally, Walling et al. reported a case of an 18-year-old female with a history of unilateral clubfoot treated with serial casting in the first two years of life [19]. The patient presented to the clinic with pain, difficulty walking, and inability to wear conventional shoes. She was treated with open Achilles tendon and flexor digitorum longus lengthening, fractional posterior tibial lengthening, midfoot capsular releases, and calcaneal and first metatarsal osteotomies. She reported satisfactory outcomes of plantigrade foot and corrected hindfoot varus [19].

Database analysis

From a total population of 25,025,699 patients in the database, 2,056 patients (0.008%) received evaluation and treatment associated with congenital clubfoot. Of these congenital clubfoot patients, 52% were male and 48% were female. The age at which these patients received any treatment associated with congenital clubfoot was further characterized (Table 3).

**Table 3:** Characteristics of congenital clubfoot-associated claims ^†^Do not sum to total because some patient records span multiple age categories. ^‡^These represent the number of claims associated with congenital clubfoot, and percentages are reported relative to the total number of such claims. Percentages may not sum to 100% due to rounding.

Characteristic	All Patients, n	Patients ≥ 20 years old, n
Total Patients	2,056	1,198
Sex		
Male	1,062 (52%)	530 (44%)
Female	994 (48%)	668 (56%)
Age^† ^ (years)		
0 to 4	597	
5 to 9	186	
10 to 14	179	
15 to 19	100	
20 to 24	31	
25 to 29	30	
30 to 34	48	
35 to 39	63	
40 to 44	79	
45 to 49	81	
50 to 54	121	
55 to 59	110	
60 to 64	110	
65 to 69	213	
70 to 74	145	
75 to 79	110	
80 to 84	48	
85 to 89	28	
Region^‡^		
Midwest	4,724 (31%)	2,207 (33%)
Northeast	200 (1%)	170 (3%)
South	8,874 (58%)	3,498 (52%)
West	1,575 (10%)	836 (12%)
Service Location^‡^		
Physician’s Office	7,228 (47%)	3,318 (49%)
Hospital Inpatient	655 (4%)	101 (2%)
Hospital Outpatient	5,273 (34%)	2,254 (34%)
Ambulatory Surgery Center	219 (1%)	177 (3%)
Other	1,998 (13%)	861 (13%)

Patients with congenital clubfoot generally received treatment for this condition in either early childhood (up to age four) or late adulthood (age: 65 to 69 years). Of these patients receiving treatment for congenital clubfoot, 1,198 (58%) were adults at the time of the treatment. Slightly more female (668 [56%]) than male (530 [44%]) congenital clubfoot patients received treatment as adults. The vast majority of treatments associated with congenital clubfoot occurred in either the office or outpatient setting for the entire cohort.

**Figure 2:**
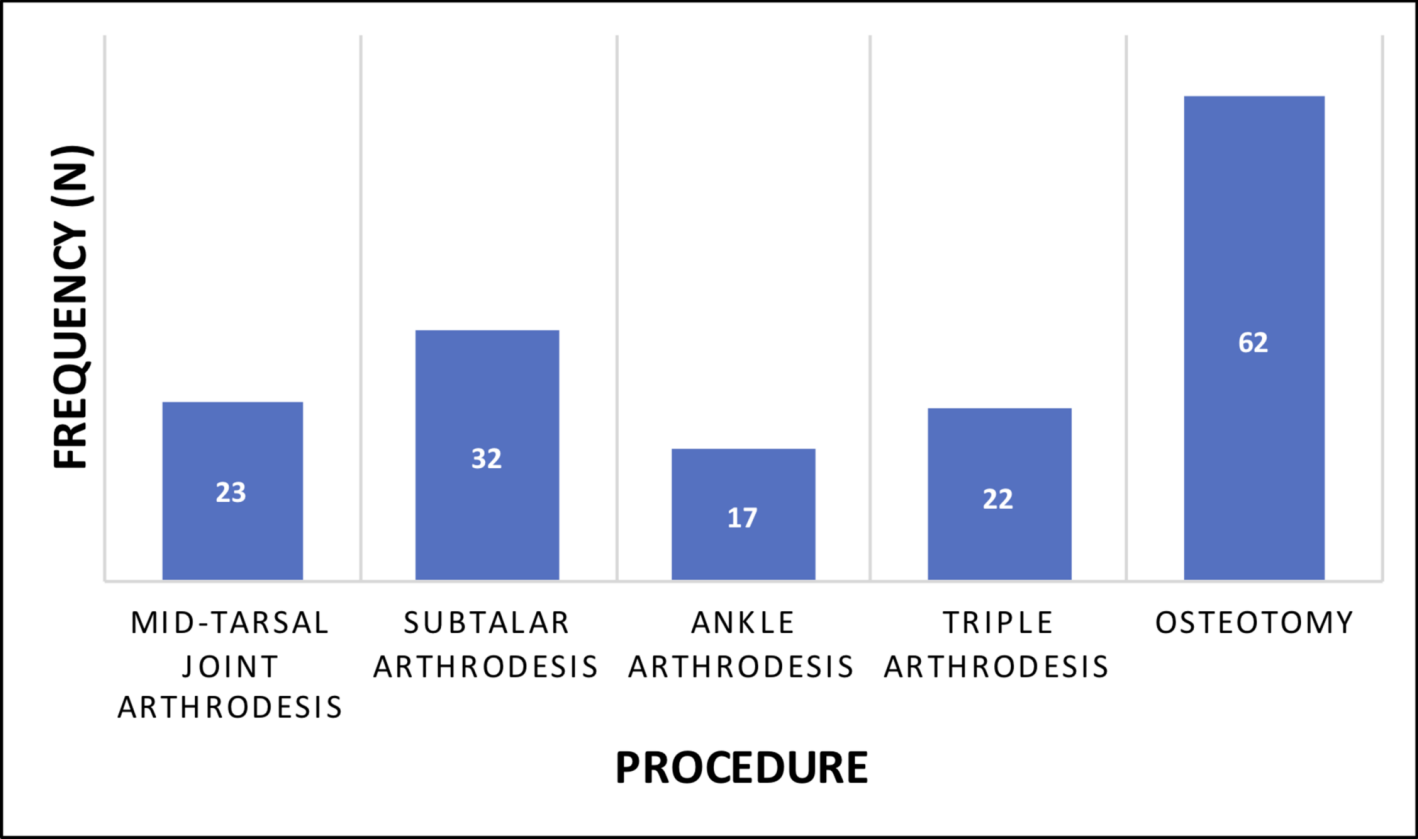
Procedures performed on adults with congenital clubfoot

Next, the frequencies of mid-tarsal joint, subtalar, ankle, and triple arthrodesis procedures and osteotomies performed in congenital clubfoot patients during adulthood were characterized (Table 4, Figure 2).

**Table 4:** Procedures performed on adults with congenital clubfoot ^†^Includes talonavicular arthrodesis. All cells for which occupancy are less than 11 have been suppressed in accordance with regulations. N/A, not applicable

Procedure	Total, n	Male, n	Female, n
Mid-tarsal joint arthrodesis^†^	23	N/A	N/A
Subtalar arthrodesis	32	11 (34%)	21 (66%)
Ankle arthrodesis	17	N/A	N/A
Triple arthrodesis	22	11 (50%)	11 (50%)
Osteotomy	62	19 (31%)	43 (69%)

There was an insufficient number of total ankle arthroplasties in this population for further analysis. The majority of adult congenital clubfoot patients (63%) who underwent mid-tarsal joint, subtalar, ankle, and/or triple arthrodesis procedures did so between the ages of 55 and 74. Similarly, the majority of adult congenital clubfoot patients (52%) who underwent osteotomy did so between the ages of 55 and 74. A total of 94 arthrodesis procedures in 73 patients were reported in the database. As 1,198 adult patients in the database received treatment associated with congenital clubfoot, 73/1,198 (6.1%) of these congenital clubfoot patients who were treated as adults received arthrodesis surgery. Similarly, 62/1,198 (5.2%) of these congenital clubfoot patients who were treated as adults received osteotomies.

## Discussion

Very few reports exist on the operative treatment of recurrent and residual clubfoot deformities in adults after initial correction during infancy. A thorough review of the literature on congenital clubfoot treatment in adults revealed that arthrodesis was cited in multiple case series, along with osteotomy. To provide further information on surgery in adult clubfoot patients, the population of congenital clubfoot patients who underwent arthrodesis and osteotomy procedures as adults were described using a large administrative claims database covering over 25 million patients. In this database, a subset of adult patients with congenital clubfoot (6.1%) underwent hindfoot arthrodesis surgery. Future work is needed to better characterize other treatments that are utilized by adult patients with congenital clubfoot and the outcomes for those who undergo ankle or foot surgeries, especially as current evidence is largely limited to case series. For example, the establishment of a longitudinal clubfoot treatment registry to track short- and long-term outcomes, severity, and further treatment or surgeries required in a standardized manner would facilitate more detailed analyses of longitudinal outcomes. Efforts to create similar registries are already ongoing in the joint replacement literature [20].

**Figure 3:**
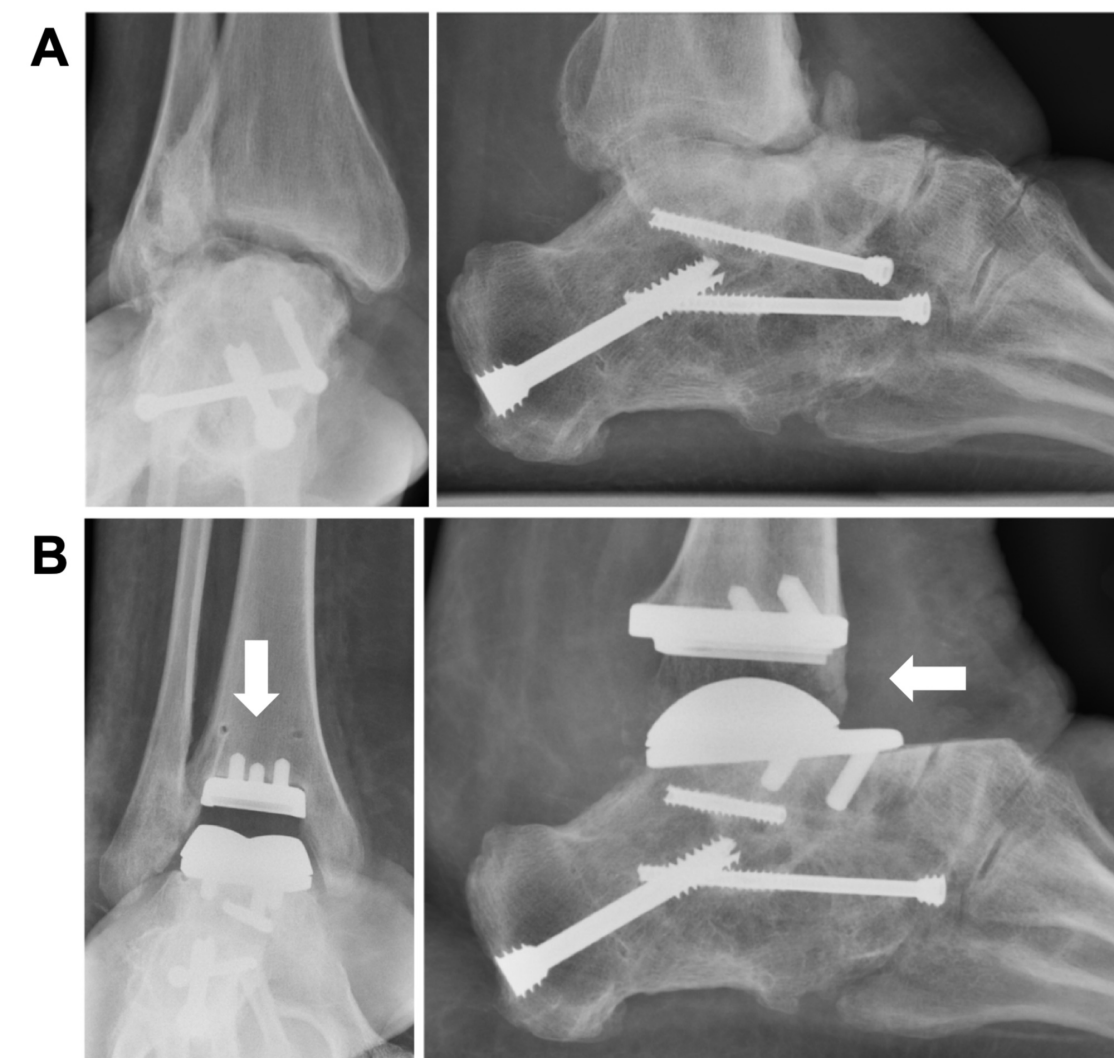
A) Preoperative and B) postoperative anteroposterior and lateral radiographs of an adult patient with previously operated clubfoot with ankle arthritis who underwent total ankle arthroplasty Ankle arthroplasty procedures (arrows) are being performed in adult clubfoot patients but are not yet reported in the literature. Case courtesy of Jeremy McCormick, MD, St. Louis, MO

Interestingly, no substantial number of adult clubfoot patients undergoing total ankle arthroplasty could be identified in the database, nor could a case report of total ankle arthroplasty in a clubfoot patient, or a patient with a diagnosis of clubfoot in reports on total ankle arthroplasty, be found in the literature review. This might be attributable to reduced preoperative subtalar mobility, which could result in a higher rate of early loosening after total ankle arthroplasty, or abnormal talar morphology and size precluding total ankle arthroplasty in clubfoot patients [21-22]. However, total ankle arthroplasty has been performed in adult patients with previously-treated congenital clubfoot (Figure 3). Thus, while total ankle arthroplasty may be an operative option for ankle arthritis in adult clubfoot patients, such cases are not yet reported in the literature and the true frequency of such procedures remains unknown. Therefore, future studies are needed to describe the efficacy and outcomes of total ankle arthroplasty performed on adult clubfoot patients.

Although the database contained 1,198 adult patients with congenital clubfoot, 94% of these patients did not undergo mid-tarsal joint arthrodesis, subtalar arthrodesis, ankle arthrodesis, triple ankle arthrodesis, total ankle arthroplasty, or osteotomies for the treatment of this condition as adults (Table 3). While likely uncommon as evidenced by our literature review, some of these patients may have undergone soft tissue procedures and thus were missed by our database analysis. Nevertheless, it remains unknown whether viable non-operative treatment modalities or surgical alternatives to hindfoot arthrodesis exist for these patients. Further outcomes research using longitudinal data is needed to answer this question.

These results should be viewed in light of the limitations. The inherent limitations of database research, including a lack of precision with coding schemes, have been reviewed elsewhere [12]. Although the database comprises the representation of all U.S. states and diverse medical and surgical specialties, it is limited due to being from a single insurer with a market share that likely differs by geographic region and thus may not be representative of the national adult clubfoot population. This may limit external generalizability. Moreover, our data only captures insured populations, with few Medicaid and no uninsured patients represented, which further limits generalizability. Further, with a prevalence of one in 1,000 live births, approximately 25,000 cases of congenital clubfoot would be expected in the database of over 25 million members. Instead, only 2,056 patients with congenital clubfoot were identified even though all diagnostic fields were queried [1]. This suggests that patients with congenital clubfoot do not commonly seek treatment related to this condition after a certain age (i.e. after age 14) and were missed by our analysis. The bimodal distribution of age within the clubfoot claims data is consistent with this interpretation (Table 3). Other explanations include inaccurate, incomplete, or incorrect medical coding, or that adult clubfoot patients are ultimately diagnosed with ankle or foot arthritis and coded as such without including the clubfoot code. Thus, the denominators within this study (e.g. total congenital clubfoot patients in the database) should be interpreted with caution. In other words, although this study demonstrates the frequency of arthrodesis and osteotomy procedures in adult clubfoot patients, the percentage of clubfoot patients treated as children who require further surgery as adults remains uncertain.

In addition, although the prevalence of congenital clubfoot is at least twice as high in males compared to females, we found that slightly more female than male clubfoot patients received treatment as adults [23]. We cannot exclude systematic differences between the clubfoot population in our database and the overall clubfoot population, especially since there is only a slight male clubfoot predominance in the entire database when children are included. However, because claims databases only capture patients who are treated, it is also possible that females are more likely to seek treatment for clubfoot as adults, perhaps due to sex-specific differences in severity and/or risk of recurrent or residual clubfoot. Notably, a recent study in infants found no difference in clubfoot severity due to sex [24]. Nevertheless, because claims databases cannot capture clubfoot severity, studies with longer-term follow-up to evaluate sex-based differences in previously-treated adult clubfoot patients are warranted.

Also, the possibility that these procedures were not performed to treat congenital clubfoot but rather for unrelated, co-morbid lower extremity conditions cannot be excluded in the database analysis. Nevertheless, the literature search showed that most surgeries performed in adult clubfoot patients were due to osteoarthritis or other degenerative changes. There is additional evidence for higher rates of osteoarthritis in adult patients treated for clubfoot in childhood. For example, a prospective study on children with unilateral clubfoot treated before the age of two and followed for 13 to 24 years showed that osteoarthritis was more severe in clubfeet than in contralateral non-clubfeet [25]. Other studies have reported arthritis, along with varus and valgus deformities and degeneration of the foot and ankle, as medical problems that patients treated for clubfoot in childhood have as adults [2,17,26]. While discussing treatment options for adult clubfoot patients, Walling stated that triple arthrodesis is the only option for stiffness and arthritis at joints following clubfoot surgery during childhood [19]. Finally, it is known that operative treatment of clubfoot in childhood, such as extensive soft tissue release, can result in several complications including osteoarthritis, often presenting as painful and stiff feet [25,27].

Finally, the clinical care of clubfoot in infants and children has changed in the past few decades, and the database analysis cannot provide information on how the patients were previously treated in childhood (i.e. nonoperatively or operatively). Thus, the question of whether successful nonoperative correction leads to a lower rate of adult ankle or foot surgery cannot be answered by the analysis of this dataset. Additionally, although the Ponseti method is currently the gold standard of care for the treatment of congenital clubfoot, some children today still receive other treatments for clubfoot and there are adult patients who were treated with other techniques as children who now have pain and/or residual deformity. Thus, it was important to elucidate the surgeries that are being performed on adults who received non-Ponseti treatments as children and now require further treatment. Future studies are warranted to explore the epidemiology, severity, and treatment methods for clubfoot in children (including the Ponseti method), following these patients through adulthood to document ankle and foot function and rates of subsequent surgery.

## Conclusions

Despite the prevalence of congenital clubfoot as a birth defect of the lower limb, little information exists on operative procedures utilized in adults with previously-treated congenital clubfoot. In this report, an extensive literature review of the operative treatments used in adults with previously-treated congenital clubfoot revealed that hindfoot arthrodesis and osteotomy have been reported in this population. This study then characterized the frequency at which adult congenital clubfoot patients underwent mid-tarsal joint arthrodesis, subtalar arthrodesis, ankle arthrodesis, triple arthrodesis, and osteotomy in a large, administrative claims database.

## Appendices

The following search strings were used in the PubMed and Embase databases, respectively.

I. (("Clubfoot/drug therapy"[Mesh] OR "Clubfoot/surgery"[Mesh] OR "Clubfoot/therapy"Mesh]) OR ((clubfoot[Mesh] OR clubfoot[tw]) AND (((ankle[tw] OR subtalar[tw] OR talonavicular[tw] OR tarsal[tw] OR tarsus[tw]) AND (fuse[tw] OR fusion[tw] OR fusing[tw] OR arthrodesis[tw] OR replacement[tw] OR arthroplasty[tw])) OR "triple arthrodesis"[tw] OR (osteotomy[tw] AND (calcaneal[tw] OR midfoot[tw] OR "first metatarsal"[tw])) OR talectomy[tw]))  OR ("surgery" [Subheading] AND clubfoot[Mesh])) AND (adult[Mesh] OR adult[tw])

II. ((('ankle fusion':ti,ab,kw OR 'subtalar fusion':ti,ab,kw OR 'talonavicular fusion':ti,ab,kw OR 'triple arthrodesis':ti,ab,kw OR talectomy:ti,ab,kw OR  'first metatarsal osteotomy':ti,ab,kw OR 'midfoot osteotomy':ti,ab,kw OR 'calcaneal osteotomy':ti,ab,kw OR 'subtalar arthrodesis'/exp OR 'subtalar arthrodesis':ti,ab,kw OR 'ankle arthroplasty'/exp OR 'ankle arthroplasty':ti,ab,kw OR 'ankle arthrodesis'/exp OR 'ankle arthrodesis':ti,ab,kw OR 'ankle replacement'/exp OR 'ankle replacement':ti,ab,kw)  AND ('clubfoot'/exp AND 'clubfoot':ti,ab,kw)) OR (('clubfoot'/exp AND 'clubfoot':ti,ab,kw) AND ('drug therapy'/lnk OR 'surgery'/lnk OR 'therapy'/lnk))) AND (adult OR 'adult'/exp)
